# Editorial: Molecular organization, evolution, and function of ribosomal DNA

**DOI:** 10.3389/fpls.2022.994380

**Published:** 2022-08-04

**Authors:** Roman A. Volkov, Nikolai Borisjuk, Sònia Garcia, Aleš Kovařík, Julio Sáez-Vásquez

**Affiliations:** ^1^Department of Molecular Genetics and Biotechnology, Yuriy Fedkovych Chernivtsi National University, Chernivtsi, Ukraine; ^2^Jiangsu Key Laboratory for Eco-Agricultural Biotechnology Around Hongze Lake and Jiangsu Collaborative Innovation Centre of Regional Modern Agriculture and Environmental Protection, School of Life Sciences, Huaiyin Normal University, Huai'an, China; ^3^Institut Botànic de Barcelona - Consejo Superior de Investigaciones Cientificas(IBB-CSIC), Barcelona, Spain; ^4^Department of Molecular Epigenetics, Institute of Biophysics, Academy of Sciences of the Czech Republic, Brno, Czechia; ^5^CNRS, Laboratoire Génome et Développement des Plantes (LGDP), UMR 5096, Perpignan, France

**Keywords:** concerted evolution, epigenetics, molecular phylogeny and taxonomy, nucleolus, polyploidy, rRNA processing

## Introduction

The aim of this Research Topic is to highlight the current status of knowledge and research on plant ribosomal DNA (rDNA). The Topic compiles seven Original Research papers, five Reviews, one Perspective and one Methods articles, viewed more than 26,000 times by the time of this Editorial. The scope covers diverse modern technologies, scientific approaches, and research aimed at achieving a better understanding of the many, complex aspects of rDNA structure, evolution, regulation, and functions in plant development and adaptation.

The rDNA encodes four ribosomal RNA (rRNAs), which are the major components of ribosome and constitute 65–75% of the plant cell's total RNA. Because of its abundance, functional importance and specific organization in evolutionarily conserved rRNA coding sequences, and rapidly evolving intergenic spacer (IGS) regions, the chromosomal and molecular organization, transcription and evolution of the rDNA have been intensively studied since the early days of plant molecular biology.

The history of rDNA research started almost 90 years ago when McClintock (1934) observed that in the interphase nuclei of maize the nucleolus was formed in association with a specific region of a chromosome, which she called the nucleolar organizer region (NOR). Early rDNA research in plants is presented in article of Hemleben et al., which covers topics such as the synthesis of rRNA precursors, processing, the organization and evolution of 5S and 18S-5.8S-26S (or 35-45S) rDNA as well as epigenetic phenomena and the impact of hybridization and allopolyploidy on rDNA expression and homogenization. This historical view sets the scene for the other articles highlighting the progress in modern rDNA research.

## Ribosomal DNA function and expression

The rRNA synthesis involves specialized transcription complexes built around RNA Polymerase I for 35-45S rRNAs and RNA Polymerase III for 5S rRNA. The regulation of rDNA expression in response to multiple internal factors and external stimuli utilizes various epigenetic mechanisms such as DNA methylation, histone modifications or RNA interference. Havlová and Fajkus focus on unusual structural features of DNA, namely shortly spaced oligo-guanine tracts able to form G-quadruplex (G4) structures. They discussed the role of these structures in regulating rDNA activity in two model plants, *Arabidopsis thaliana* (angiosperm) and *Physcomitrella patens* (moss).

Nucleolar dominance (ND) represents the selective silencing of parental 35-45S rDNA loci in the genome of a hybrid or allopolyploid. Borowska-Zuchowska et al. analyzed ND in two genotypes of a model allotetraploid grass, *Brachypodium hybridum* (Poaceae). They found that ND was developmentally stable in one but not the other accession, the latter showing a codominant expression of parental rDNA in adventitious roots.

The current knowledge on the 35-45S pre-rRNA modifications, which is a mandatory step of rRNA processing during ribosome assembly in the nucleolus, is summarized by Streit and Schleiff. Hundreds of ribosome biogenesis factors (RBFs) and small nucleolar RNAs (snoRNA) catalyze the rRNA processing (cleavages and modifications) in *Arabidopsis thaliana*. Small nucleolar ribonucleoprotein particles (snoRNPs) complexes, composed of C/D or H/ACA snoRNAs and RBFs, catalyze, respectively, the two major rRNA modification types, 2'-*O*-ribose-methylation and pseudouridylation, which are required for stability of rRNA structure and for translational accuracy and efficiency.

Coordinated production and integration of rRNA and protein components into cytoplasmic ribosomes and the nucleolar organizer regions (NORs) in response to changes in genetic constitution, biotic and abiotic stresses are reviewed by Appels et al. Using a hexaploid wheat *Triticum aestivum* (Poaceae) as a model, it is argued that unique functionalities of ribosome populations can become central in situations of stress conditions by preferentially translating mRNAs coding for proteins contributing to survival of the cell.

## Organization and evolution of rDNA

5S and 35-45S rDNA are composed of numerous copies of tandemly arranged repeated units (repeats), which are usually located on one or few chromosomal loci. In many species, numerous rDNA repeats tend to be very similar due to sequence homogenization, i.e., individual repeats do not evolve independently, but in a concerted manner (Arnheim et al., 1980; Coen et al., 1982; Volkov et al., 1999). The level of intragenomic homogenization may differ in different taxa and for the different rDNAs (5S vs 35-45S), and the reason for this remains enigmatic. Sims et al. provided a hypothetical model under which the genetic landscape of rDNA arrays is driven by a balance between non-homologous end joining (NHEJ) and homologous recombination (HR). While NHEJ increases the array heterogeneity by introducing point mutation and indels, HR acts in the opposite direction homogenizing the units. It is widely believed that the rDNA repeats should be nearly identical within the same chromosomal locus, while the repeats from different loci may show lower similarity. This idea was confirmed by examining diploid and polyploid species of the genus *Rosa* (Rosaceae), for which two highly divergent 5S rDNA families located on different chromosomes were identified (Vozárová et al.). Both gene families arose in the early history of the genus, already 30 myrs ago, and apparently survived numerous speciation events thereafter.

The intragenomic diversity of 5S rDNA was examined for 137 *Solanum* (Solanaceae) species (Tynkevich et al.), possessing one 5S locus per chromosome set. It was shown that many repeat variants coexist within the genome demonstrating incomplete sequence homogenization. The main mechanisms of 5S rDNA molecular evolution in the genus *Solanum* was step-wise accumulation of single base substitution or short insertions/deletions (indels) in the 5S IGS, whereas long indels and multiple base substitutions were mostly not conserved and eliminated.

In this Research Topic, the molecular organization of rDNA is explored for the first time for the aquatic plant duckweed *Landoltia punctata* representing the Lemnaceae. Chen et al. demonstrated the presence of two classes of 5S rDNA repeats, which differ by the composition and distribution of subrepeats in the IGS, and regulatory DNA elements potentially involved in 5S rDNA transcription. The genome of *L. punctata* has one of the lowest copy numbers of rDNA genes among flowering plants and an unusual, mosaic arrangement of 5S rDNA clusters. Stepanenko et al. characterized rDNA of another aquatic species, *Pistia stratiotes*, from the Araceae family. Whereas, the 5S and 35-45S rDNA were localized in a single chromosome locus each, the species' 35-45S rDNA is represented by at least four length variants, distinguished by the number of subrepeats within the IGS. The 5S rDNA locus includes at least six types of functional gene units, intermingled with each other and with pseudogenes. The data support the idea of a mosaic arrangement of multiple variants of 5S and 35-45S rDNA units in single locus as the rule rather than the exception.

Nuclear rDNA demonstrates extraordinary dynamics during evolution. In diploid *Hordeum* (Poaceae) species, Krak et al. analyzed the fate of alien 35-45S rDNA copies acquired *via* horizontal transfer from panicoid genera. The foreign ribotypes were present in the respective genomes at low copy numbers, likely representing a minor fraction of the total rDNA dedicated to pseudogenization.

It is clear that the knowledge of the intragenomic diversity of 35-45S rDNA is still incomplete, since large NORs are generally missing from existing genome assemblies due to their highly repetitive nature. In the future, organization of these complex areas composed of relatively long (~9–20 kb) regions can potentially be deciphered by third generation sequencing methods, such as Oxford Nanopore Technology (ONT). This issue is addressed by McKinlay et al., who developed a method providing enrichment of 35-45S rDNA sequences among ultra-long ONT reads.

## Taxonomic application of rDNA

The rapidly evolving spacer regions of rDNA provide a convenient tool for phylogenetic studies of lower-ranking taxa. Fehrer et al. point out that the appropriate treatment of intra-individual variation and the investigation of multiple markers allows interesting insights in complex species relationships as well as in the evolution of the markers themselves. In the *Hieracium* (Asteraceae) genus, they found that chromosomal location of the 5S and 35-45S rDNA loci is far more dynamic than the sequences they contain, implying that chromosomal patterns are not suitable to infer species relationships, at least not in *Hieracium*.

The comparison of 5S IGS was successfully applied to reconstruct the phylogeny of the giant genus *Solanum* (Solanaceae) (Tynkevich et al.), allowing clarification of taxonomic position of several species and detection of reticulate evolution, especially in its largest section, *Petota*.

The correct interpretation of rDNA markers in plant taxonomy and evolution is not free of drawbacks. Accordingly, Rosselló et al. aim to discuss the limitations of nuclear 35-45S rDNA markers based on cytological and karyological experimental work to draw sound biological and evolutionary conclusions in a systematic and phylogenetic context. The authors offer clarification for some misconceptions usually found in published work that could help lead to an insightful utilization of the ribosomal DNA world in plant evolution.

## Author contributions

The manuscript was written by RV with inputs from NB, SG, JS-V, and AK. All authors approved the submitted version.

## Funding

RV and AK thank respectively the Ministry of Education and Science of Ukraine (Grant No. 0122U001335) and the Czech Science Foundation (20-14133J) for support. NB was supported by an individual grant provided by the Huaiyin Normal University (Huai'an, China).

## Conflict of interest

The authors declare that the research was conducted in the absence of any commercial or financial relationships that could be construed as a potential conflict of interest.

## Publisher's note

All claims expressed in this article are solely those of the authors and do not necessarily represent those of their affiliated organizations, or those of the publisher, the editors and the reviewers. Any product that may be evaluated in this article, or claim that may be made by its manufacturer, is not guaranteed or endorsed by the publisher.
